# Genome-wide analysis of *Ariadne* genes associated with drought and salt stress tolerance in wheat (*Triticum aestivum* L.)

**DOI:** 10.1186/s12870-026-09416-7

**Published:** 2026-07-04

**Authors:** Zilin Fan, Xin Sun, Yuyi Ma, Xu Lu, Xinxin Hong, Xuning Liu, Xun Long, Fa Cui, Hao Liu, Xiaoyu Wang

**Affiliations:** 1 Yantai Key Laboratory of Molecular Breeding for High-Yield and Stress-Resistant Crops and Efficient Cultivation/ College of Horticulture, Ludong University, Yantai, 264025 China; 2 College of Fisheries, Ludong University, Yantai, 264025 China; 3 College of Chemical and Biological Engineering, Shandong University of Science and Technology, Qingdao, 266590 China; 4 Yantai Puxiang Agricultural Development Co., Ltd., Yantai, 264000 China

**Keywords:** Ariadne, Drought and salt stress, Expression patterns, Wheat

## Abstract

**Background:**

The *Ariadne* (*ARI*) gene family belongs to RBR-type E3 ubiquitin ligases and regulates plant growth, development, and abiotic stress responses. However, a systematic genome-wide analysis of the *ARI* family has not been reported in wheat.

**Results:**

In this study, we identified 21 *TaARI* genes that were unevenly distributed on 12 chromosomes. The phylogenetic analysis showed that ARI proteins were divided into A, B, and C subfamilies. Most TaARIs existed in subfamilies A and B, and only TaARI7-D belonged to subfamilies C. TaARIs with closer phylogenetic relationships had similar gene structures and conserved motifs. Promoter *cis*-acting element analysis revealed that stress-responsive elements were the most abundant in the promoter of *TaARI*s. Expression profiling demonstrated that most *TaARI*s were preferentially expressed in roots, and induced by PEG-simulated drought or salt stress, with *TaARI2*, *TaARI3*, *TaARI4*, *TaARI5* and *TaARI6* as candidate stress regulators for drought and salt stresses. A total of 72 upstream transcription factors of *TaARI*s belonging to 24 transcription factor families were determined, with MYB-related, bZIP, and ERF transcription factors being the most abundant. Protein interaction prediction indicated that TaARIs interact with E3 ubiquitin-protein ligase RBX1 and ubiquitin-40 S ribosomal protein S27a, suggesting that TaARIs might participate in the ubiquitin–proteasome pathway. Haplotype analysis suggested that *TaARI6-D-Hap I* could be a superior drought-resistant haplotype with higher gene expression and seedling survival under drought stress.

**Conclusions:**

In conclusion, our results provided crucial insights into the functions and molecular mechanisms of *TaARI* genes under drought and salt stress, and provided a gene resource for molecular breeding to stress‑resistant wheat varieties.

**Supplementary Information:**

The online version contains supplementary material available at https://doi.org/10.1186/s12870-026-09416-7.

## Introduction

As one of the three major staple crops in the world, wheat (*Triticum aestivum*) occupies an irreplaceable position in ensuring food security. However, wheat has to suffer various abiotic stresses in actual production, such as drought, high temperature, and saline-alkali stresses, hindering the growth and development of wheat and significantly reducing yield, which has become a key bottleneck restricting the stable development of the wheat industry [1–3]. Therefore, identification of stress-resistant genes and breeding of stress-resistant cultivars are important for improving wheat yield and grain quality.

*ARI* (*Ariadne*) family genes belong to a subgroup of RBR-type (RING between RING fingers) E3 ubiquitin ligase, and play central roles in plant growth and development, hormone signaling, and abiotic stress response through ubiquitination-mediated protein degradation [4–6]. As RBR-type E3 ubiquitin ligase, ARI proteins exhibit acidic enrichment amino acid residues in the N-terminus, a leucine enrichment region and a coiled helix domain in the C-terminus, and a conserved RING1-IBR-RING2 domain (RBR domain) in the middle region [4, 7, 8]. Among the RBR domains, the RING1 domain is responsible for binding to the E2 enzyme, the RING2 domain mediates ubiquitin transfer, and the IBR domain maintains protein conformational stability; all of them synergistically ensure the E3 ligase activity [8–10].

The *ARI* genes have been functionally characterized in several plant species, such as *Arabidopsis thaliana* [11], apple (*Malus domestica*) [4], *Brassica napus* [5], and soybean (*Glycine max*) [6, 7]. In *Arabidopsis*, a total of 16 *AtARI* genes were identified [11]. Among them, *AtARI12* was regulated by bZIP transcription factors HY5 and HYH, and involved in UV‑B signaling and photomorphogenesis through interaction with Constitutively Photomorphogenic 1 (COP1) [12, 13]. In *Hypericum perforatum*, *HpARI*, the orthologous gene of *AtARI7*, was identified that related to apospory [14]. In apple, *MdARI*s exhibited a significant response to drought and salt stresses, and MdARI7-2 interacted with E2-conjugating enzyme (MdUBC11a and MdUBC11b), suggesting that MdARI7-2, as an E3 ubiquitin ligase participated in plant growth and stress response processes [4]. In addition, heterologous overexpression of soybean *GmARI1* enhanced aluminum resistance of *Arabidopsis*, further supporting the conserved function of the *ARI* gene in plant stress resistance [6].

Although *ARI* genes were identified in several plant species, a systematic genome‑wide analysis of the *ARI* gene family in wheat has not been reported. In this study, we identified and comprehensively analyzed wheat *ARI* family members, including their phylogenetic relationships, chromosomal distribution, gene structures, conserved motifs, promoter *cis*‑elements, tissue‑specific expression, stress‑responsive patterns, protein interactions, upstream transcriptional regulators, and haplotypes. Our findings provide insights into the evolutionary and functional characteristics of wheat *TaARI* genes and identify key candidates for improving drought and salt tolerance in wheat molecular breeding.

## Materials and methods

### Genome-wide identification of *ARI* family genes in wheat

The reference genome, protein sequences and GFF3 annotation file of wheat were obtained from EnsemblPlants database (http://plants.ensembl.org/index.html). In order to identify candidate *ARI* genes in wheat, the Hidden Markov Model (HMM) of Ariadne domain (PF19422) and IBR domain (PF01485) (http://pfam.xfam.org) was used to search against the wheat protein sequences through TBtools [15]. Meanwhile, candidate *ARI* genes were also retrieved through Blastp search against wheat protein sequences with 16 AtARI protein sequences [11]. All candidate *ARI* genes were validated using Batch CD-Search (https://www.ncbi.nlm.nih.gov/Structure/bwrpsb/bwrpsb.cgi) and the InterPro (https://www.ebi.ac.uk/interpro/) websites. The physicochemical properties of TaARIs were calculated using the Protein Parameter Calc in TBtools software [15]. Subcellular localization prediction was performed using the WoLF PSORT website (https://wolfpsort.hgc.jp/).

### Multiple sequence alignment and phylogenetic tree construction

The ARI protein sequences from *A. thaliana*, rice (*O. sativa*), apple (*M. domestica*), and wheat (*T. aestivum*) were used for multiple sequence alignment with ClustalW and phylogenetic tree construction with Maximum Likelihood (ML) method in MEGA11 under default parameters [4, 11, 16, 17]. Then, the phylogenetic tree of ARI proteins was visualized by the Evolview service (https://www.evolgenius.info/evolview-v3/) [18].

### Chromosomal localization and collinearity analysis

Chromosomal localization information was retrieved from GFF3 annotation file of wheat genome, and intraspecific collinearity and gene duplication events were detected using the One Step MCScanX - Super Fast, and visualized by Advanced Circos in TBtools software [15]. The nonsynonymous substitution rate (Ka), synonymous substitution rate (Ks) and Ka/Ks ratio of paralogous *TaARI* gene pairs were calculated to evaluate the evolutionary selection pressure with the Nei-Gojobori (NG) method in TBtools [15].

### Gene structure and conserved motifs analysis of *TaARI* genes

The gene structure information was obtained from the GFF3 annotation file. The conserved motifs of the TaARIs protein family were analyzed using the MEME website (https://meme-suite.org/meme/) with the motif number parameter set to 10 [19]. The conserved domains were obtained from the Batch CD-Search (https://www.ncbi.nlm.nih.gov/Structure/bwrpsb/bwrpsb.cgi). The visualization of gene structures, conserved motifs, and domains were performed using the Gene Structure View (Advanced) function in TBtools software [15].

### The *cis*-acting elements analysis of *TaARI*s promoters

The 2 kb promoter sequences of *TaARI* genes were obtained from EnsemblPlants database (http://plants.ensembl.org/index.html), and then the PlantCare website (http://bioinformatics.psb.ugent.be/webtools/plantcare/html/) was used to analyze the distribution and quantity of *cis*-acting elements in the promoters.

### The upstream transcription factors analysis of *TaARI*s

The upstream transcription factors of *TaARI*s were obtained through wGRN database (https://wheat.cau.edu.cn/wGRN/). The transcriptome data of wheat under drought (BioProject ID: PRJNA1003680) and salt stresses (BioProject ID: PRJNA632706) were used to analyze the co-expression clustering of *TaARI*s and their upstream transcription factors, and visualized by TBtools software [15].

### Protein interaction analysis of wheat ari gene family

The interacting proteins of TaARIs were retrieved through the String database (https://cn.string-db.org/). The correlation of expression under drought and salt stresses between TaARIs and interacting proteins were analyzed based on the transcriptome data (BioProject ID: PRJNA1003680 and PRJNA632706), and the correlation heatmap was drawn by the OmicShare platform [20].

### qRT-PCR assay

The 7-day-old seedlings of wheat cultivar “Chinese Spring” with uniform leaf size and root length were selected to determine the expression levels of *TaARI*s in different tissues and stress conditions by qRT-PCR. To determine the expression levels of *TaARI*s in different tissues, the shoot and root tissues of 7-day-old seedlings were collected. For drought and salt stresses, the 7-day-old seedlings were cultured with 20% PEG6000 (w/v) and 300 mM NaCl treatments, and the leaf tissues were collected at 0, 12, 24, and 36 h, respectively. All collected samples were frozen in liquid nitrogen and stored at − 80 °C for further qRT-PCR assay.

Total RNA extraction from samples was performed using the FastPure Universal Plant Total RNA Isolation Kit (Vazyme), and reverse transcription was conducted with the Hifair^®^V one-step RT-gDNA digestion SuperMix for qPCR kit (Yeasen). A qRT-PCR assay was carried out using the SGExcelFast SYBR Mixture (Sangon Biotech) reagent kit. The qRT-PCR primers of *TaARI*s were shown in Table S1, and *TaActin* gene was selected as a reference control for normalization. All reactions were performed on three replicates, and the relative expression level was calculated using the 2^−ΔΔCt^ method [21].

### Drought-resistant superior haplotype analysis

The haplotypes of the *TaARI* gene were analyzed using resequencing data of 2789 hexaploid wheat cultivars from the WheatUnion database (https://wheat.cau.edu.cn/WheatUnion/). The expression levels of *TaARI* genes in 200 wheat cultivars under well-watered (WW) and drought stress (DS) conditions were analyzed using the transcriptome data (CRA016347) [22], which obtained from the Genome Sequence Archive of the National Genomics Data Center (https://bigd.big.ac.cn/gsa). The survival rates of 400 wheat cultivars under drought stress were obtained from published data [23], and then the survival rates of wheat cultivars harboring different haplotypes of *TaARI* gene were analyzed. The correlation analysis between survival rate and expression levels of *TaARI* genes in different wheat cultivars after well-watered and drought stress treatments were analyzed based on the above the survival rates and transcriptome data.

## Results

### Identification and physicochemical properties analysis of *ARI* gene family

**Fig. 1:**
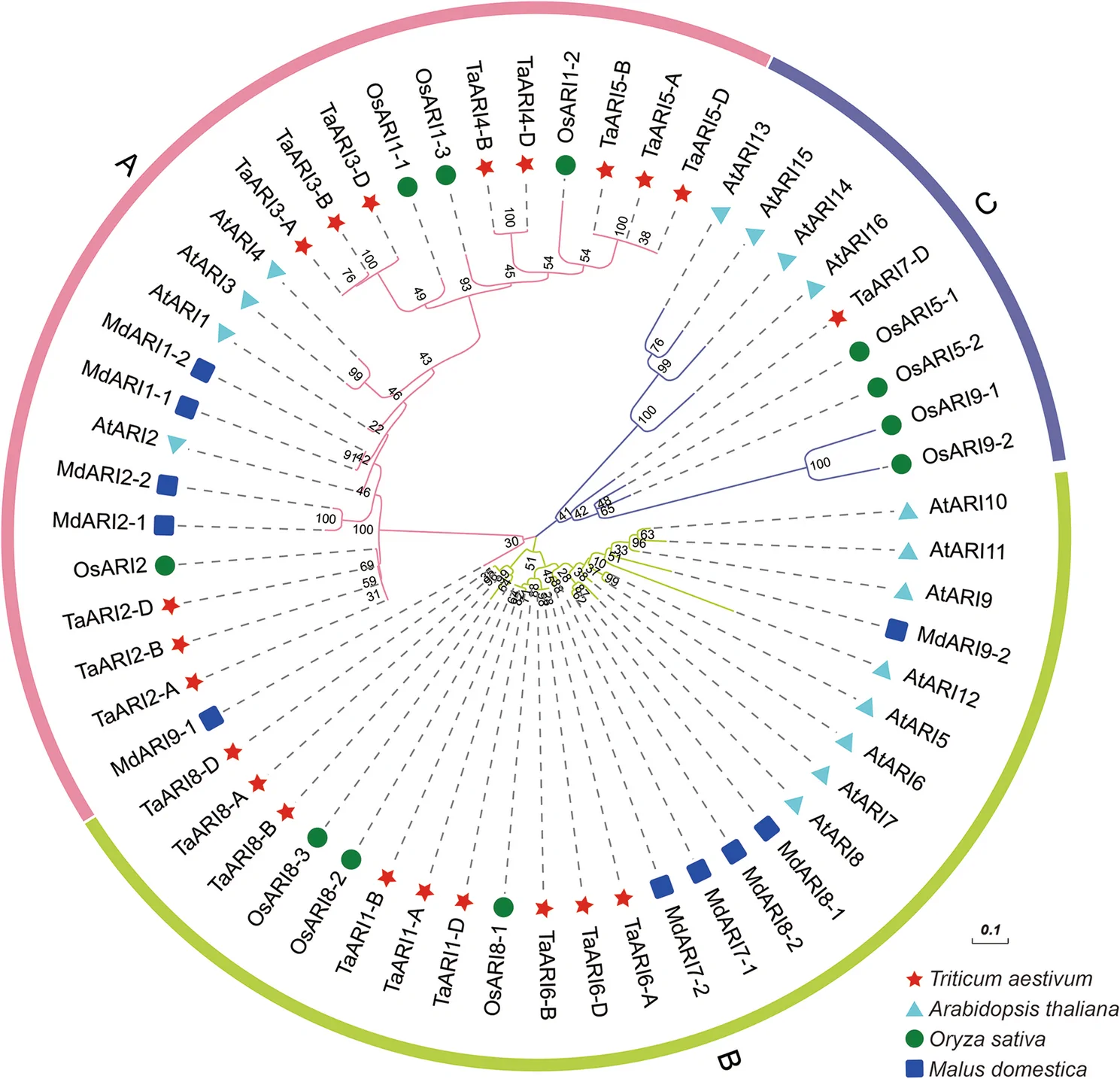
Phylogenetic tree of ARI proteins in different plant species. The ARI protein sequences from *T. aestivum* and *A. thaliana*, *O. sativa*, and *M. domestica* were used to construct the phylogenetic tree with Maximum Likelihood (ML) method and 1000 bootstrap replicates. The ARI protein from *T. aestivum* and *A. thaliana*, *O. sativa*, and *M. domestica* sequences were marked with different symbols

Based on the Hidden Markov Model (HMM) search against the wheat genome, a total of 21 *ARI* genes were identified in the wheat. According to their chromosomal positions and evolutionary relationships, these *TaARI* genes were named as *TaARI1-A/B/D* to *TaARI8-A/B/D* (Table S2 and Fig. 1). These 21 *TaARI* genes encoded 491 to 620 amino acid residues, with molecular weights ranging from 55.5 to 70.9 kDa. The isoelectric point (pI) values varied from 4.94 to 5.98, suggesting TaARI proteins were acidic proteins. The Grand average of hydropathicity (GRAVY) values were negative, suggesting that all TaARI proteins are hydrophilic. Subcellular localization prediction indicated that all TaARI proteins localize in the nucleus.

### Phylogenetic relationship of TaARIs

To clarify evolutionary relationships of TaARIs, a phylogenetic tree was constructed using 21 wheat TaARIs, 16 *Arabidopsis* AtARIs, 11 rice (*Oryza sativa*) OsARIs, and 10 apple MdARIs (Fig. 1 and Table S3). The ARIs were divided into three subfamilies, i.e., A, B, and C subfamilies. Most TaARIs were distributed in subfamilies A and B, and only TaARI7-D belonged to subfamily C. TaARI2‑A/B/D, TaARI3‑A/B/D, TaARI4‑B/D, and TaARI5‑A/B/D belonged to subfamily A, and TaARI1‑A/B/D, TaARI6‑A/B/D and TaARI8‑A/B/D were clustered into subfamily B. Among them, TaARI3‑A/B/D, TaARI4‑B/D and TaARI5‑A/B/D showed high homology with OsARI1‑1, OsARI1‑3 and OsARI1‑2, respectively. TaARI2‑A/B/D exhibited high homology with OsARI2, TaARI1‑A/B/D and TaARI6‑A/B/D were closely related to OsARI8-1, TaARI8‑A/B/D were closely related to OsARI8-3, TaARI7‑D shared high homology with OsARI9-1 and OsARI9-2. These results suggested that TaARI proteins might have a similar function with the homologous protein in other plant species, and provided insights for the further functional study of *TaARI* genes.

### Chromosomal localization and collinearity analysis of *TaARI* genes

**Fig. 2:**
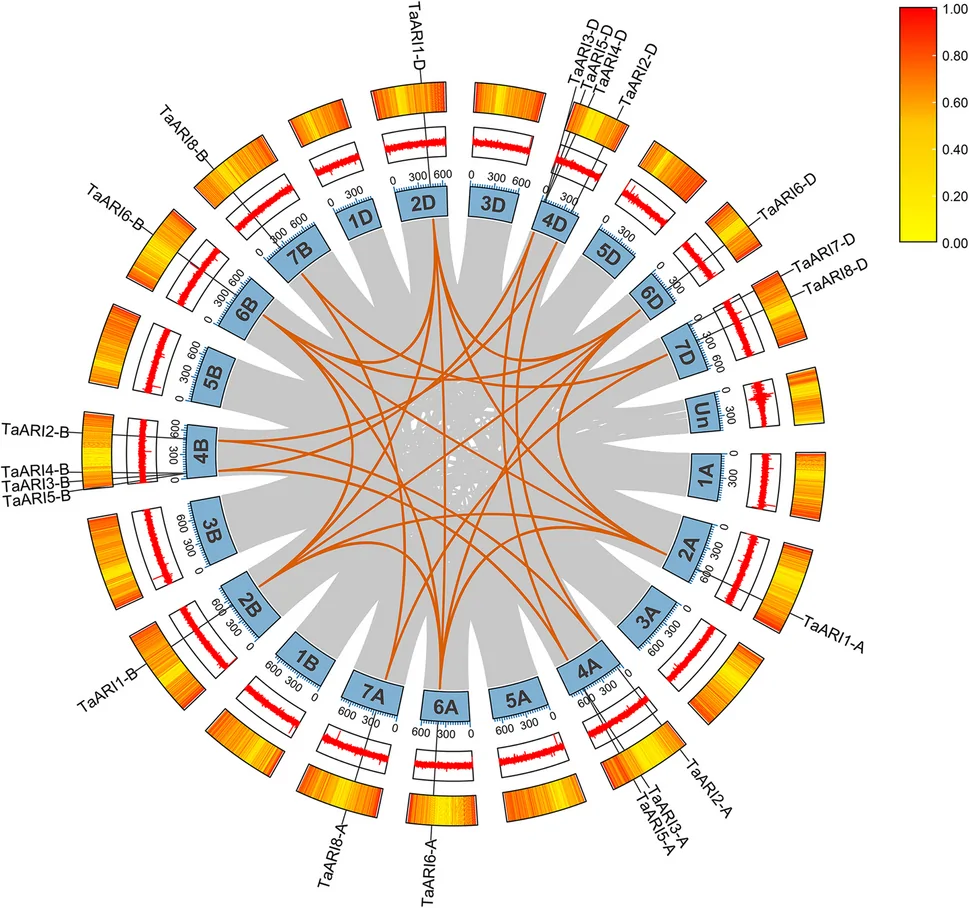
Chromosome localization and collinearity analysis of the *TaARI* genes among *T. aestivum.* Tracks from inside to outside showed chromosomes, GC content, and gene density. The lines in the circle represented the collinearity relationships of wheat genes, and the red lines indicated the collinearity relationships of *TaARI* genes

The chromosomal localization result indicated that 21 *TaARI* genes were unevenly distributed across twelve chromosomes, i.e., chromosome 2 A, 2B, 2D, 4 A, 4B, 4D, 6 A, 6B, 6D, 7 A, 7B, and 7D (Fig. 2 and Table S2). Chromosomes 4B and 4D contained four *TaARI* genes which were the two largest number of *TaARI* genes on chromosome, followed by chromosome 4 A with three *TaARI* genes, and 7D with two *TaARI* genes, chromosomes 2 A, 2B, 2D, 6 A, 6B, 6D, 7 A, and 7B contained one gene, respectively. All 21 *TaARI* genes undergone WGD or segmental events (Table S2). The synteny analysis revealed that twenty-four paralogous gene pairs were detected among *TaARI* genes, such as *TaARI1-A/B/D*, *TaARI2-A/B/D*, *TaARI3-A/B/D*, *TaARI4-B/D*, *TaARI5-A/B/D*, *TaARI6-A/B/D*, and *TaARI8-A/B/D* among different subgenomes (Fig. 2 and Table S4). Additionally, *TaARI1-A/B/D* and *TaARI6-A/B/D* exhibited synteny. The Ka/Ks values of these twenty-four paralogous gene pairs were less than 1 (Table S4), suggesting that these paralogous gene pairs undergone purifying selection during the evolutionary process to maintain the function of *TaARI* gene family.

### Gene structures and conserved motifs analysis of *TaARI* genes

**Fig. 3:**
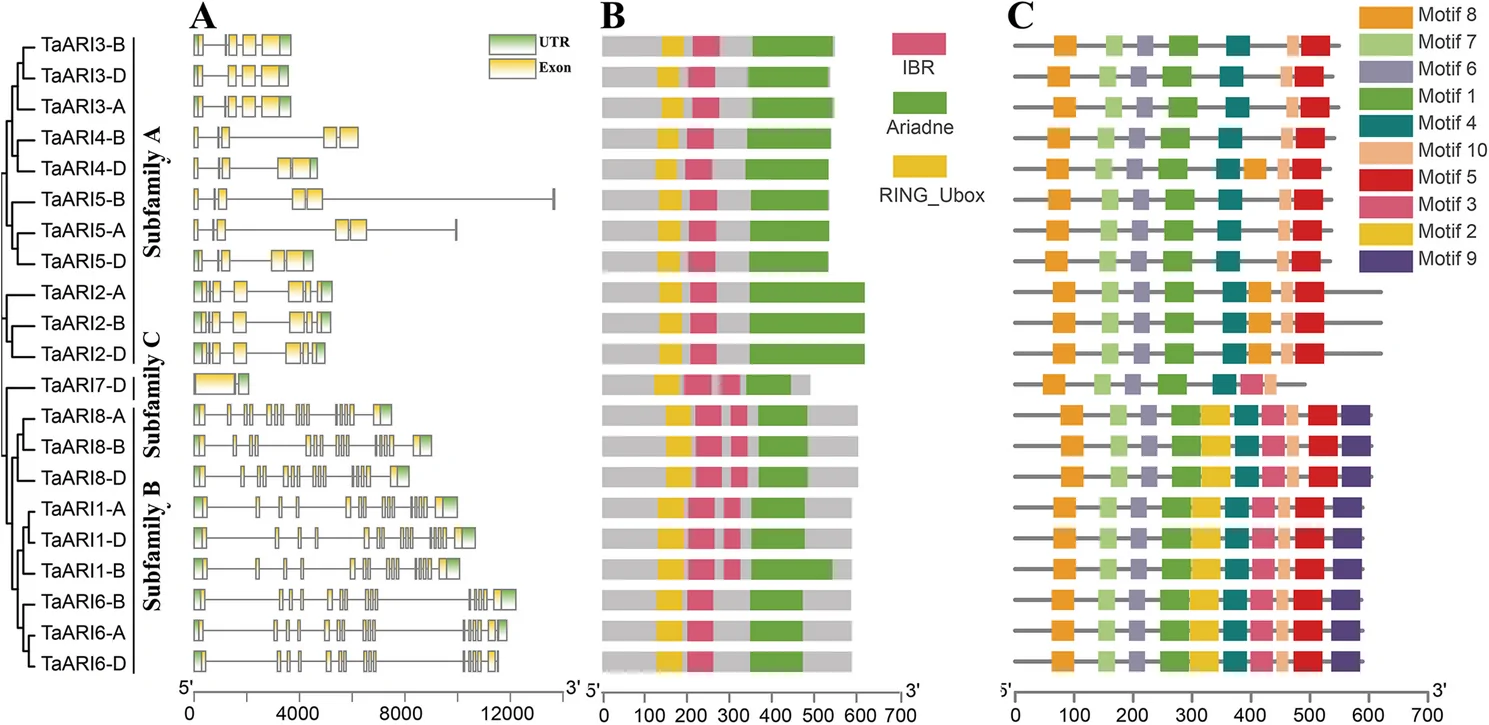
Gene structure and conserved motifs of TaARIs. The exon-intron structures **A**, domains **B** and conserved motifs **C** of TaARIs were shown

To investigate the structural characteristic of *TaARI* genes, the gene structures and conserved motifs were analyzed (Fig. 3). The results showed that *TaARIs* of subfamily A contained 5–7 exons, The subfamily A members *TaARI2-A*, *TaARI2-B*, *TaARI2-D*, *TaARI3-A*, *TaARI3-B*, *TaARI3-D*, *TaARI4-B*, *TaARI4-D*, *TaARI5-A*, *TaARI5-B* and *TaARI5-D* included 7, 7, 7, 5, 5, 4, 5, 5, 6, 6 and 5 exons. Most members of the subfamily B contained 15–16 exons, and the subfamily C member *TaARI7-D* contained one exon (Fig. 3A and Table S2). The results of conserved motifs suggested that all TaARIs contained one Ariadne domain, one RING_Ubox domain, and at least one IBR domain. Among them, only TaARI1-A/B/D, TaARI7-D and TaARI8-A/B/D included two IBR domains (Fig. 3B). Ten conserved motifs of TaARIs (motif 1–10) were also detected (Fig. 3C). All TaARIs protein included motif 4, 5, 6, 7, 8 and 10, indicating that they were the core functional motifs. Motif 2, 3 and 9 were unique to the B subfamily, suggesting that might contribute to subfamily-specific functions (Fig. 3C and Table S2). These results suggesting that TaARIs with closer phylogenetic relationships had similar gene structures and conserved motifs (Fig. 3).

### Analysis of *cis*-acting elements in *TaARI* promoters

**Fig. 4:**
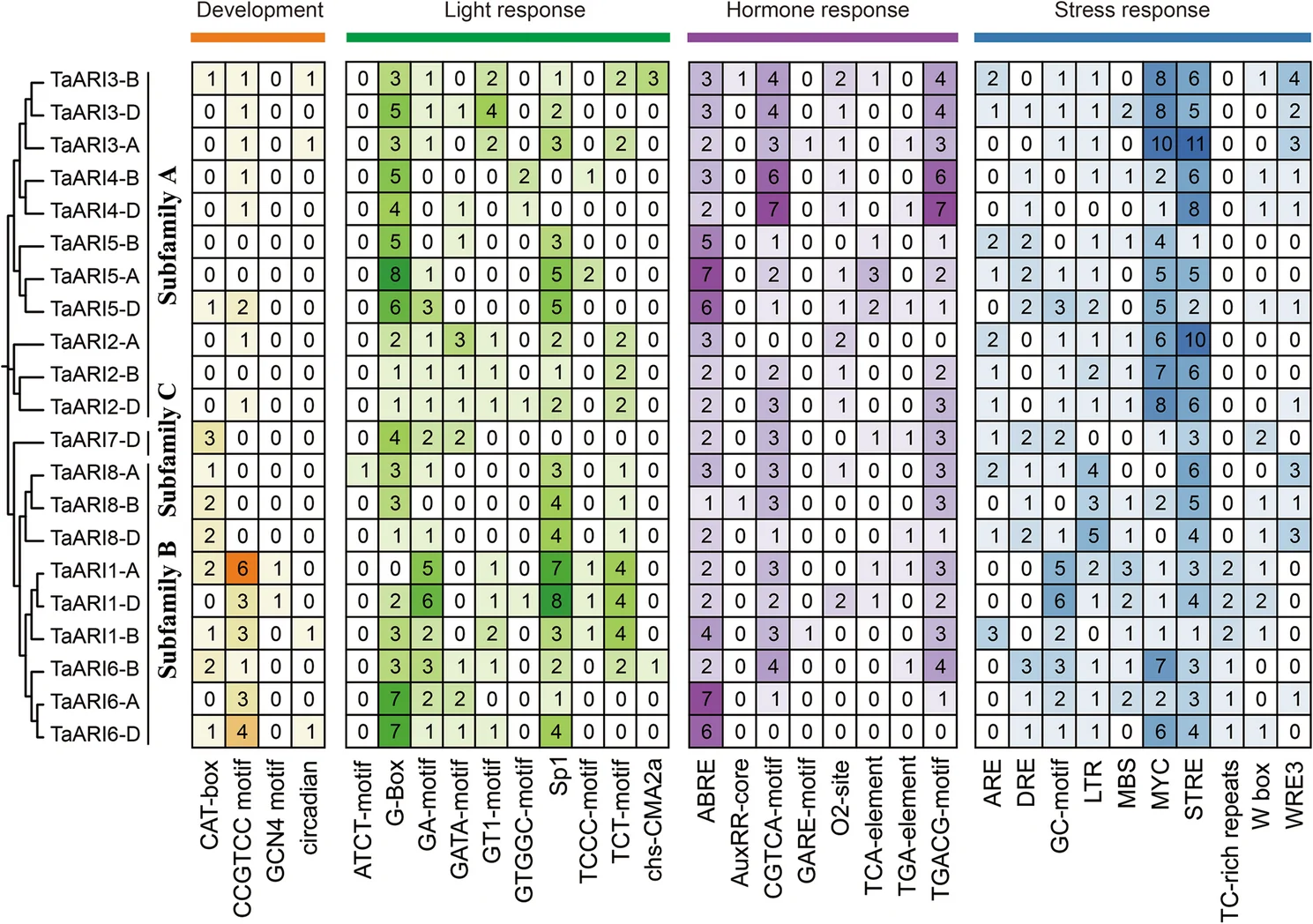
Analysis of *cis*-acting elements in *TaARI* promoters. The different colors and numbers of grids indicated the counts of *cis*-acting elements in *TaARI* promoters

A total of 32 types of *cis*-acting elements related to development, light response, hormone response, and stress response were detected in the promoters of *TaARI*s (Fig. 4 and Table S5). Notably, stress-responsive elements were the most abundant with 355 *cis*-acting elements in the promoter regions of *TaARI*s, especially abundant in subfamily B members. Among them, STRE and MYC elements were the predominant *cis*-acting elements, suggesting a central role of *TaARI*s in mediating responses to environmental stressors. Light-responsive elements represented the second largest category with 217 *cis*-acting elements, including 75 G-Box and 49 Sp1 *cis*-acting elements, indicating potential involvement in light signaling and photomorphogenesis. Hormone-responsive elements were also highly abundant with 206 *cis*-acting elements, containing 69 ABRE (ABA-responsive element), 55 CGTCA-motif and 53 TGACG-motif elements as the most frequent elements, suggesting regulation of *TaARI*s by ABA (abscisic acid) and MeJA (methyl jasmonate) signaling pathways. The development-related elements mainly contained meristem expression elements (CAT-box) and CCGTCC motifs, implying *TaARI*s might participate in plant growth and development processes. These results demonstrated that *TaARI* genes might respond to stress, light and hormones, and function as key integrators of environmental stress and endogenous hormones in wheat.

### Tissue-specific and stress-responsive expression patterns of *TaARI*s

**Fig. 5:**
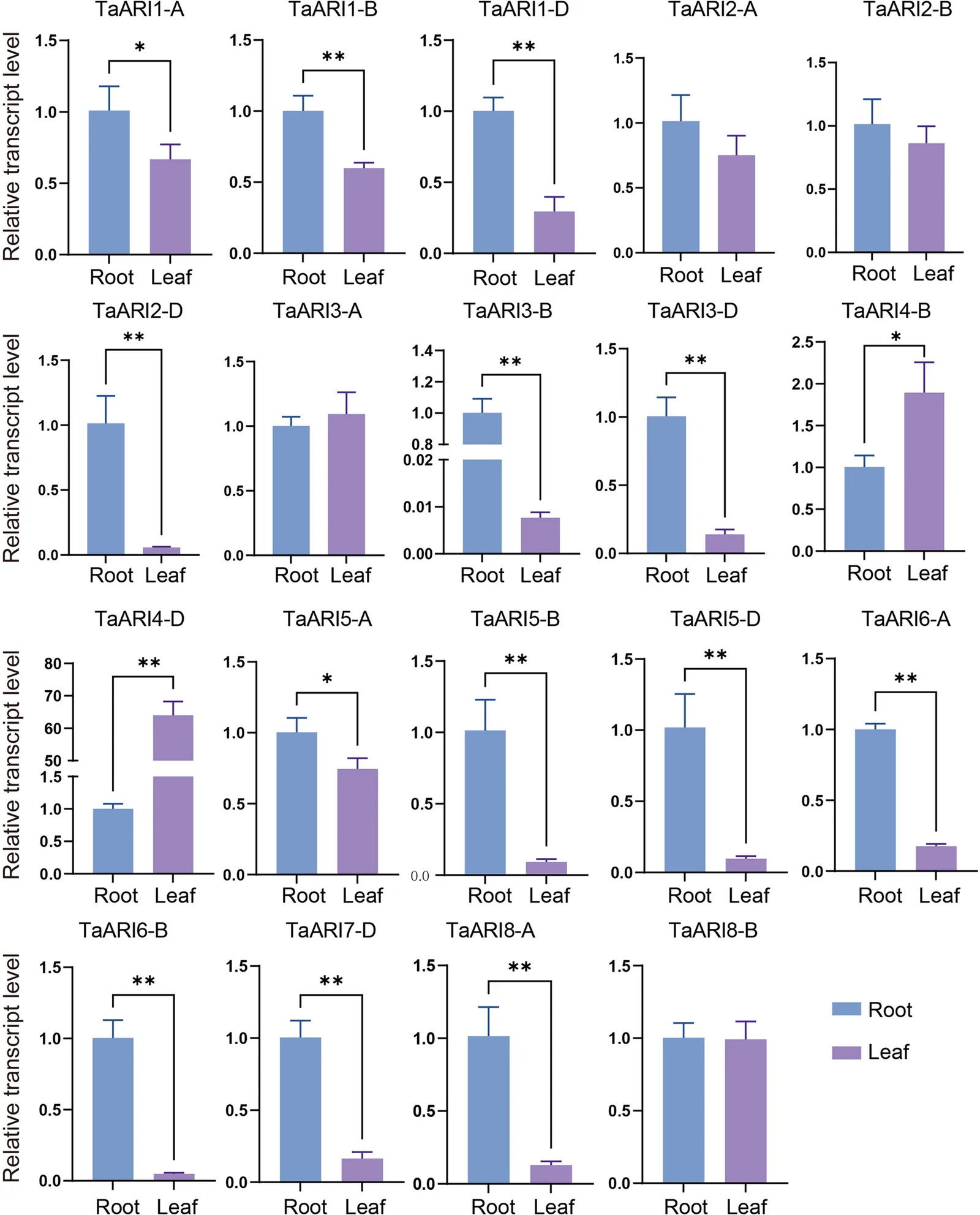
Expression patterns of *TaARI* genes in leaves and roots. qRT-PCR was performed to detect the expression patterns of *TaARI* genes in leaf and root tissues at seedling stage. The values displayed the mean ± SD. Significant statistical analysis was conducted using Student’s t-test (* *p* < 0.05; * * *p* < 0.01)

**Fig. 6:**
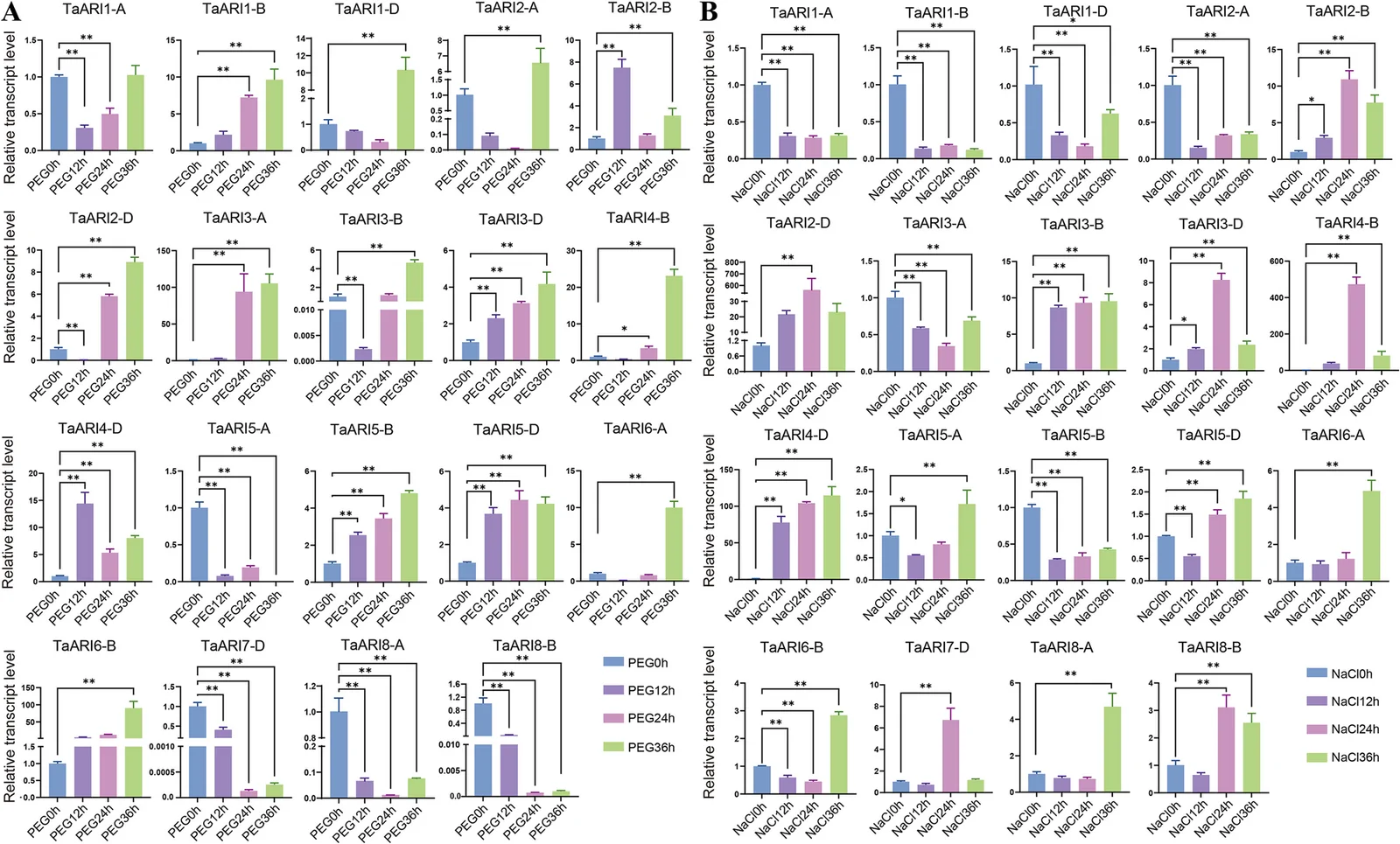
Expression patterns of *TaARI* genes under drought and salt stresses. qRT-PCR was performed to detect the expression patterns of *TaARI* genes under PEG‑simulated drought **A** and salt **B** stresses in wheat seedling leaves. The values displayed the mean ± SD. Significant statistical analysis was conducted using Student’s t-test (* *p* < 0.05; * * *p* < 0.01)

**Fig. 7:**
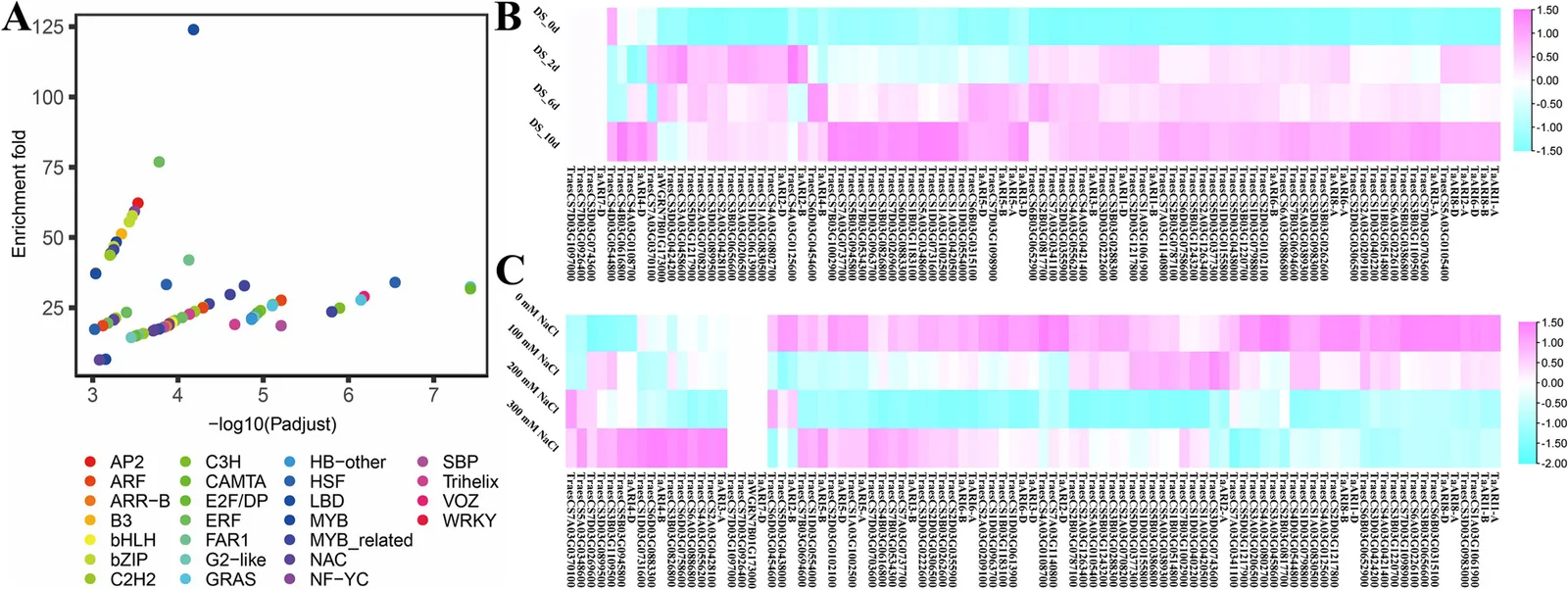
The upstream transcription factors analysis of *TaARI* genes. **A** The upstream transcription factor predication of *TaARI*s through wGRN database. **B** Expression patterns of TFs and *TaARI*s under drought stress based on RNA-seq. **C** Expression patterns of TFs and *TaARI*s under salt stress based on RNA-seq

In order to investigate the function of *TaARI*s, quantitative real-time PCR (qRT-PCR) was performed to detect the expression patterns of *TaARI*s in leaf and root tissues, and PEG‑simulated drought and salt stress treatments at seedling stage (Figs. 5, 6 and 7). qRT-PCR results showed the expression levels of *TaARI1-A/B/D*, *TaARI2-D*, *TaARI3-B/D*, *TaARI5-A/B/D*, *TaARI6-A/D*, *TaARI7-D* and *TaARI8-A* were significantly higher in roots compared with leaves. *TaARI4-B/D* were significantly down-regulated in roots compared to leaves. The expression levels of *TaARI2-A/B*, *TaARI3-A*, *TaARI8-B* had no obvious change in root and leaves (Fig. 5).

After drought stress treatment, the most *TaARI* genes exhibited significant up-regulated expression compared with control, including *TaARI1-B*/*D*, *TaARI2-A/B/D*, *TaARI3-A/B/D*, *TaARI4-B/D*, *TaARI5-B/D*, *TaARI6-A/B*. In contrast, *TaARI7-D* and *TaARI8-A/B* were significantly down-regulated under drought stress (Fig. 6A). Under salt stress, the expression levels of *TaARI2-B/D*, *TaARI3-B/D*, *TaARI4-B/D*, *TaARI5-A/D*, *TaARI6-A/B*, *TaARI7-D* and *TaARI8-A/B* were significantly up-regulated compared with control, and *TaARI1-A/B/D*, *TaARI2-A*, *TaARI3-A* and *TaARI5-B* exhibited significant down-regulated expression (Fig. 6B). These results suggesting that *TaARI2*, *TaARI3*, *TaARI4*, *TaARI5* and *TaARI6* might be candidate stress regulators for drought and salt stresses (Fig. 6).

### Analysis of upstream transcription factors of *TaARI* genes

The upstream transcription factors of the wheat *TaARI* genes were detected through wGRN database, and a total of 72 upstream transcription factors of *TaARI*s belonging to 24 transcription factor (TF) families were determined, with MYB-related, bZIP and ERF transcription factors being the most abundant (Fig. 7 and Table S6). To explore the regulatory mechanism of *TaARI*s under drought and salt stresses, the co-expression clustering of *TaARI*s and their upstream transcription factors were analyzed based on transcriptome data (Fig. 7, Fig. S1 and Table S7). Under drought stress, the most similar expression patterns were identified between *TaARI*s with TFs, i.e., *TaARI1-A* co-clustered with GRAS (*TraesCS5A03G0389300*), *TaARI1-B* co-clustered with G2-like (*TraesCS3B03G0288300*), *TaARI1-D* co-clustered with G2-like (*TraesCS3D03G0222600*), *TaARI2-A* and *TaARI2-B* co-clustered with MYB-related (*TraesCS5A03G0105400*), *TaARI2-D* co-clustered with VOZ (*TraesCS1A03G0830500*), *TaARI3-A* co-clustered with NAC (*TraesCS2A03G0209100*) and ARF (*TraesCS1D03G0402200* and *TraesCS1B03G0514800*), *TaARI3-B* co-clustered with ERF (*TraesCS6D03G0758600*), *TaARI3-D* co-clustered with MYB (*TraesCS1D03G0731600*), *TaARI5-A* co-clustered with MYB (*TraesCS1D03G0731600*) and NAC (*TraesCS2A03G0209100*), *TaARI5-B* co-clustered with ERF (*TraesCS1D03G0554000*), *TaARI5-D* co-clustered with bZIP (*TraesCS2D03G0306500*) and ERF (*TraesCS6D03G0758600*), *TaARI6-A* co-clustered with CAMTA (*TraesCS2B03G0787100*), MYB-related (*TraesCS7A03G1140800*) and NAC (*TraesCS2A03G0209100*), *TaARI6-B* co-clustered with CAMTA (*TraesCS2B03G0787100*) and MYB-related (*TraesCS7A03G1140800*), *TaARI6-D* co-clustered with CAMTA (*TraesCS2D03G0355900*) and G2-like (*TraesCS3D03G0222600* and *TraesCS5D03G0438000*), *TaARI8-A* co-clustered with ERF (*TraesCS3B03G0262600*), *TaARI8-B* co-clustered with bHLH (*TraesCS7B03G0694600*), *TaARI8-D* co-clustered with C3H (*TraesCS2D03G1217800*), MYB-related (*TraesCS3D03G0899500*), HSF (*TraesCS5D03G1217900*), C3H (*TraesCS3B03G0656600*), SBP (*TraesCS1D03G0613900*) and VOZ (*TraesCS1A03G0830500*) (Fig. S1).

Under salt stress, *TaARI1-A* co-clustered with Trihelix (*TraesCS1A03G1061900*), *TaARI1-B* co-clustered with HB-other (*TraesCS3D03G0983000*), *TaARI1-D* co-clustered with ARR-B (*TraesCS4A03G0125600*), *TaARI2-A* co-clustered with C2H2 (*TraesCS6B03G0652900*) and VOZ (*TraesCS1A03G0830500*), *TaARI2-B* co-clustered with E2F/DP (*TraesCS2B03G0817700*) and HSF (*TraesCS5D03G1217900*), *TaARI2-D* co-clustered with NAC (*TraesCS2D03G0102100*), *TaARI3-A* co-clustered with ERF (*TraesCS6A03G0886800*), *TaARI3-B* co-clustered with bZIP (*TraesCS2D03G0306500*), *TaARI3-D* co-clustered with NAC (*TraesCS2A03G0209100*), *TaARI5-A* co-clustered with NAC (*TraesCS2A03G0209100*), *TaARI5-B* co-clustered with ERF (*TraesCS1D03G0554000*), *TaARI5-D* co-clustered with bZIP (*TraesCS2D03G0306500*), *TaARI6-A* co-clustered with CAMTA (*TraesCS2B03G0787100*), *TaARI6-B* co-clustered with CAMTA (*TraesCS2B03G0787100*), *TaARI6-D* co-clustered with SBP (*TraesCS1D03G0613900*), *TaARI8-A* co-clustered with C3H (*TraesCS3B03G0656600*) and HSF (*TraesCS6B03G0315100*), *TaARI8-B* co-clustered with C3H (*TraesCS2D03G1217800*), *TaARI8-D* co-clustered with C3H (*TraesCS3B03G0656600*) (Fig. S1). These results provided the potential TFs regulating the expression of *TaARI*s under drought and salt stress.

### The interacting protein analysis of TaARIs

**Fig. 8:**
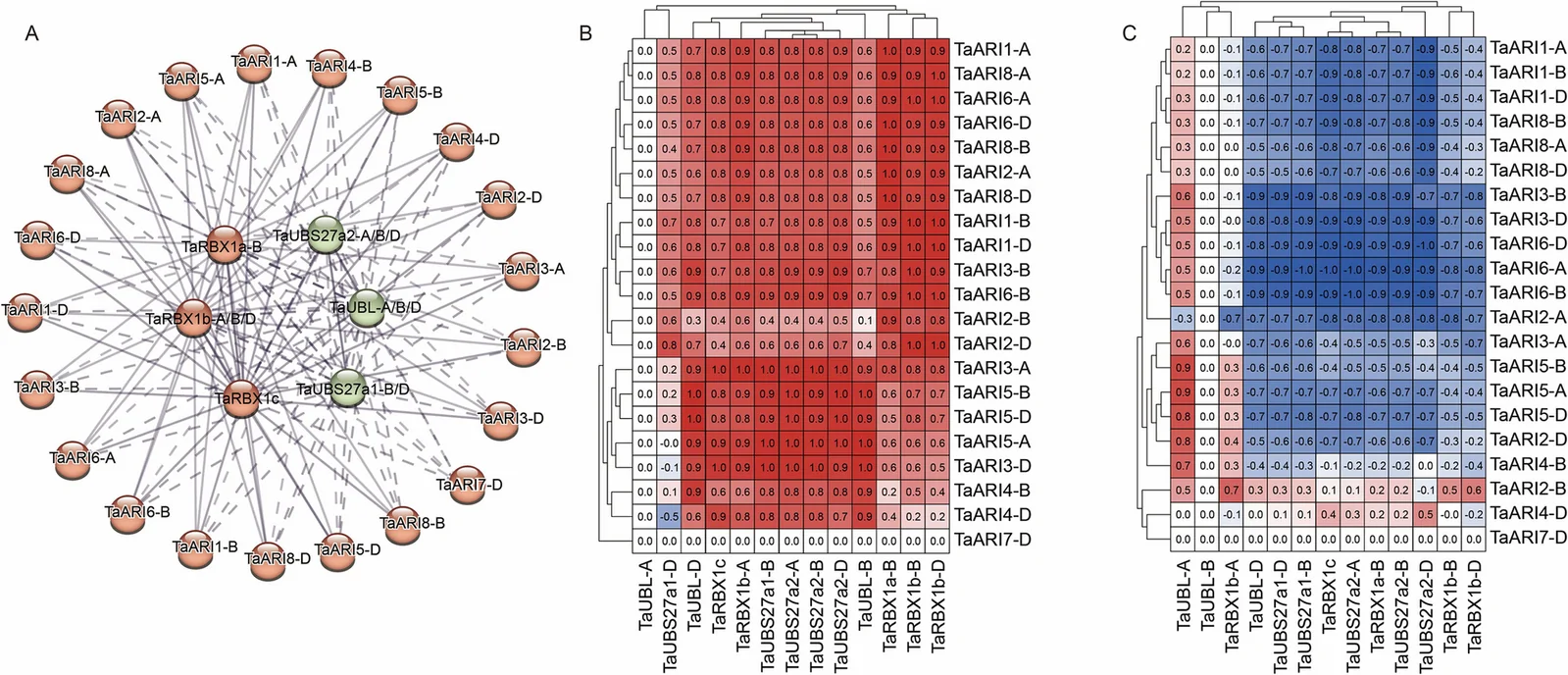
The interacting protein analysis of TaARIs. **A** The interacting protein of TaARIs were predicted through String database. **B**-**C** The correlation analysis between TaARIs and the interacting proteins at the expression level under drought **B** and salt **C** stresses based on transcriptome data

The String database was used to analyze the interacting protein of TaARIs, the results showed that all TaARIs proteins could interact with E3 ubiquitin-protein ligase RBX1 (TaRBX1a-B, TaRBX1b-A/B/D and TaRBX1c) and ubiquitin-40 S ribosomal protein S27a (TaUBL-A/B/D, TaUBS27a1-B/D and TaUBS27a2-A/B/D) (Fig. 8 and Table S8). In order to investigate potential mechanism of TaARIs under drought and salt stresses, the correlation of expression between TaARIs and interacting proteins were analyzed based on transcriptome data (Fig. 8). The results showed that TaARI1-A/B/D, TaARI2-A/B/D, TaARI3-A/B/D, TaARI4-B/D, TaARI5-A/B/D, TaARI6-A/B/D and TaARI8-A/B/D exhibited positive correlation with E3 ubiquitin-protein ligase RBX1 (TaRBX1a-B, TaRBX1b-A/B/D and TaRBX1c) and ubiquitin-40 S ribosomal protein S27a (TaUBL-B/D, TaUBS27a1-B/D and TaUBS27a2-A/B/D) at the expression level under drought stress (Fig. 8B). TaARI1-A/B/D, TaARI2-A/D, TaARI3-A/B/D, TaARI4-B, TaARI5-A/B/D, TaARI6-A/B/D and TaARI8-A/B/D were negatively correlated with E3 ubiquitin-protein ligase RBX1 (TaRBX1a-B, TaRBX1b-B/D, and TaRBX1c) and ubiquitin-40 S ribosomal protein S27a (TaUBL-D, TaUBS27a1-B/D, and TaUBS27a2-A/B/D) at the expression level under salt stress (Fig. 8C). These results suggested that TaARIs might participate in ubiquitin-proteasome pathway to regulate the growth and development of wheat under drought and salt stresses.

### Drought-resistant superior haplotype analysis of *TaARI6-D*

**Fig. 9:**
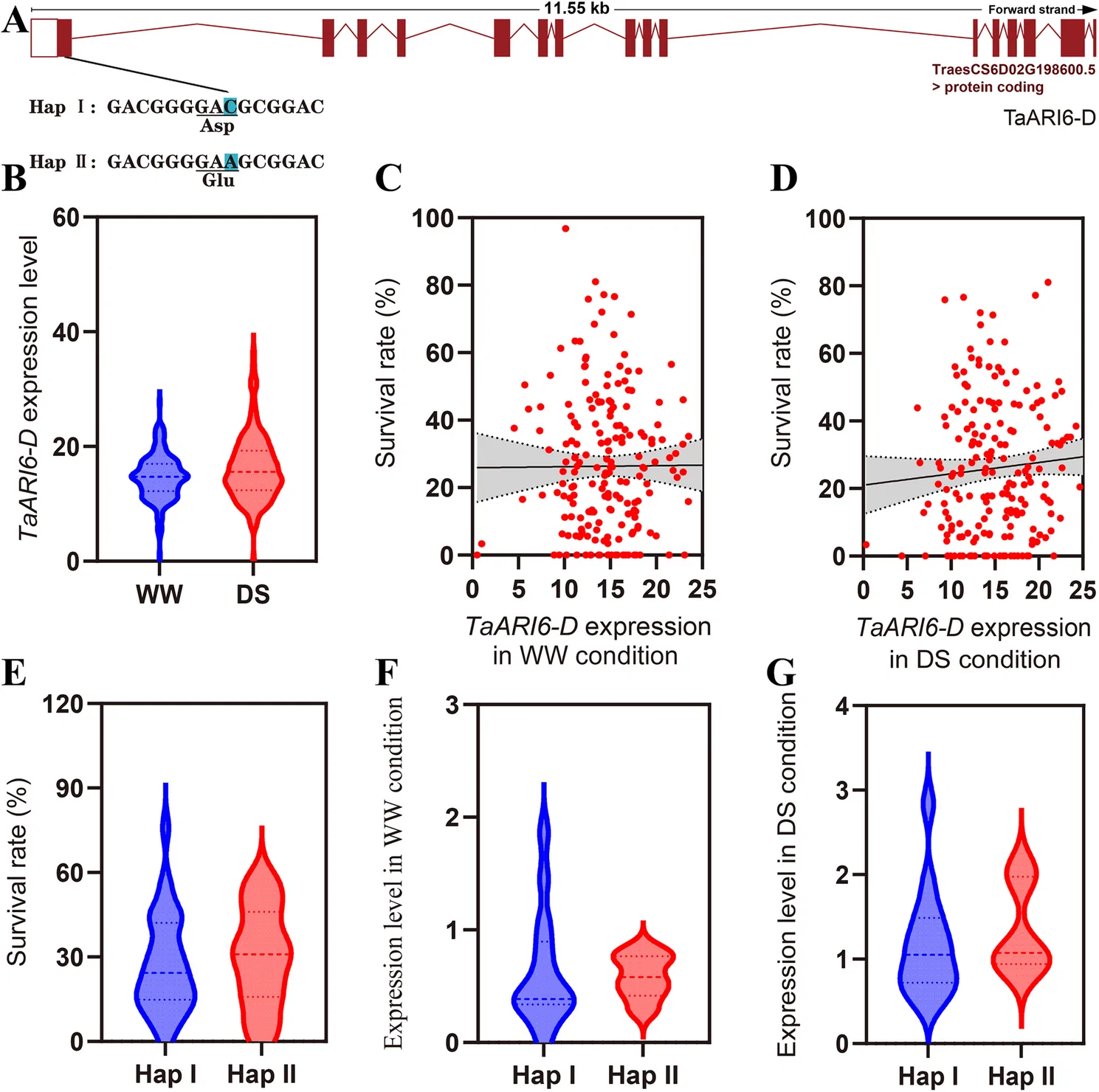
Drought-resistant superior haplotype analysis of *TaARI6-D*. **A** The main haplotype of *TaARI6-D*. **B** The expression level of *TaARI6-D* under well-watered (WW) and drought stress (DS) in 200 wheat varieties. **C**-**D** The correlation analysis between survival rate and expression level of *TaARI6-D* in WW **C** and DS **D** conditions. **E**-**G** The survival rate **E** and expression level in WW **F** and DS **G** conditions of *Hap I* and *Hap II*

The expression level of *TaARI6-D* was obviously up-regulated under drought stress (DS) compared with well-watered (WW) condition based on transcriptome data analysis of 200 wheat cultivars (Fig. 9). The survival rate of wheat seedling was positively correlated with the expression of *TaARI6-D* under drought stress, suggesting that *TaARI6-D* could be induced under drought stress, and enhanced drought resistant of wheat seedling (Fig. 9D). The haplotype analysis showed *TaARI6-D* mainly existed two haplotypes, i.e., *TaARI6-D-Hap I* and *TaARI6-D-Hap II*. The wheat cultivars carrying *Hap II* had higher survival rate and expression level of *TaARI6-D* compared with wheat cultivars carrying *Hap I* under drought stress (Fig. 9E-G). These results suggested that *TaARI6-D-Hap I* was a superior drought-resistant haplotype, providing a valuable genetic resource for breeding drought-tolerant wheat.

## Discussion

Ubiquitination is a highly conserved post‑translational modification that regulates protein stability, activity and degradation, and plays essential roles in plant growth, development, and stress responses [6, 24]. As a subgroup of RBR-type E3 ubiquitin ligases, the *Ariadne* gene family is defined by a conserved RING1‑IBR‑RING2 domain, which is critical for E2 binding and ubiquitin transfer [24]. The *ARI* genes have been characterized in several plant species, with 16 *AtARI*s in *Arabidopsis* [11], 10 *MdARI*s in apple [4], 39 *BnARI*s in *Brassica napus* [5], and 7 *GmARI*s in soybean [6, 7]. In this study, a total of 21 *TaARI* genes were identified in wheat (Fig. 1). The expansion of *ARI* gene in wheat was consistent with the characteristic of wheat allohexaploid (AABBDD), which has undergone two rounds of natural polyploidization and whole‑genome duplication during evolution process [25, 26]. All *TaARI* genes undergone WGD or segmental events, and Ka/Ks values of paralogous gene pairs among *TaARI*s were less than 1, suggesting the function of *TaARI* genes were relatively conserved (Fig. 2 and Table S4). These results were similar with other family genes in wheat, such as *ABCG* [27], *GPAT* [28], and *YUCCA* family genes, etc. TaARIs within the same subfamily showed similar gene structures and conserved motifs, while distinct differences were observed between subfamilies, suggesting functional conservation and divergence of TaARIs were occurred during evolution (Fig. 3). These structural characteristics of TaARIs were consistent with those of ARIs reported in apple and Arabidopsis [4, 11].

*ARI* genes have been reported to respond to various abiotic stresses [6, 12, 13]. In apple, all *MdARI*s significantly responded to PEG‑simulated drought and salt stresses [4]. In our study, most *TaARI* genes also exhibited significant up-regulated expression under drought and salt stresses (Fig. 6), suggesting that *TaARI* genes might play important roles in drought and salt stresses. The promotes of *TaARI*s exhibited abundant stress-responsive elements, e.g., STRE and MYC elements, which also verified the function of *TaARI*s associated with drought and salt stresses (Fig. 4). Tissue‑specific expression analysis showed that *ARI* genes, such as *TaARI*s (Fig. 5) and *BnARI*s [5], were preferentially expressed in roots, implying that *TaARI*s might participate in early stress signal perception and transduction.

*ARI* gene belonged to E3 ubiquitin ligase, and could mediate protein ubiquitination and degradation through ubiquitin-proteasome pathway [4–6]. In apple, MdARI7-2 was reported that interacted with E2-conjugating enzyme (MdUBC11a and MdUBC11b) [4]. TaARIs were predicted to interact with E3 ubiquitin-protein ligase RBX1 and ubiquitin-40 S ribosomal protein S27a (Fig. 8), and E3 ubiquitin-protein ligase RBX1 has been reported to respond to water-deficit stress [29], suggesting TaARIs participated in the ubiquitin-proteasome pathway in response to water-deficit stress. Meanwhile, the upstream transcription factors of *TaARI*s were predicted, and MYB‑related, bZIP and ERF TFs were the most abundant (Fig. 7). In *Arabidopsis*, bZIP transcription factors HY5 and HYH could regulate the expression of *AtARI12* [12, 13]. Furthermore, the co-expression clustering of *TaARI*s and their upstream TFs was analyzed to identify the key TFs potentially regulating the expression of *TaARI*s under drought and salt stress. For example, the most similar expression profiles under drought stress were detected between *TaARI3-D* and its upstream transcription factor MYB (*TraesCS1D03G0731600*), suggesting that MYB (*TraesCS1D03G0731600*) might regulate the expression of *TaARI3-D* under drought stress (Fig. 7 and Table S7). Moreover, we also identified the drought-resistant superior haplotype of *TaARI* genes, Wheat cultivars carrying *TaARI6‑D-Hap I* exhibited significantly higher expression and seedling survival rate under drought stress, suggesting that *TaARI6‑D-Hap I* was a superior drought‑resistant haplotype (Fig. 9). Collectively, this study systematically characterized *TaARI* genes in wheat, established a basis for further functional investigation of *TaARI* genes in response to drought and salt stresses, and provided valuable gene resources for molecular breeding of stress‑resistant wheat varieties.

## Supplementary Information

Supplementary Material 1: File S1. The co-expression clustering of TaARIs and their transcription factors under drought and salt stress.

Supplementary Material 2: Table S1. Specific primers used in the study. Table S2. The characteristics of ARI genes in wheat. Table S3. ARI proteins used in the phylogenetic tree construction. Table S4. Paralogous gene pairs of TaARIs in T. aestivum. Table S5. The cis-elements analysis in the promoter region of TaARI genes. Table S6. Identification of upstream transcription factors of the TaARI genes. Table S7. TPM values of TaARIs and their transcription factors under drought and salt stress. Table S8. TPM values of TaARI and thei interacting proteins under drought and salt stress.

## Data Availability

The wheat cultivar “Chinese Spring” was saved by Ludong University. The publicly available transcriptome data (BioProject ID: PRJNA1003680 and PRJNA632706) were used in this study. The transcriptome data of 200 wheat cultivars under well-watered and drought conditions were obtained from the Genome Sequence Archive of the National Genomics Data Center (CRA016347).
